# Adolescents’ Functional Numeracy Is Predicted by Their School Entry Number System Knowledge

**DOI:** 10.1371/journal.pone.0054651

**Published:** 2013-01-30

**Authors:** David C. Geary, Mary K. Hoard, Lara Nugent, Drew H. Bailey

**Affiliations:** 1 Department of Psychological Sciences, University of Missouri, Columbia, Missouri, United States of America; 2 Interdisciplinary Neuroscience Program, University of Missouri, Columbia, Missouri, United States of America; 3 Department of Psychology, Carnegie Mellon University, Pittsburgh, Pennsylvania, United States of America; George Mason University/Krasnow Institute for Advanced Study, United States of America

## Abstract

One in five adults in the United States is functionally innumerate; they do not possess the mathematical competencies needed for many modern jobs. We administered functional numeracy measures used in studies of young adults’ employability and wages to 180 thirteen-year-olds. The adolescents began the study in kindergarten and participated in multiple assessments of intelligence, working memory, mathematical cognition, achievement, and in-class attentive behavior. Their number system knowledge at the beginning of first grade was defined by measures that assessed knowledge of the systematic relations among Arabic numerals and skill at using this knowledge to solve arithmetic problems. Early number system knowledge predicted functional numeracy more than six years later (ß = 0.195, p = .0014) controlling for intelligence, working memory, in-class attentive behavior, mathematical achievement, demographic and other factors, but skill at using counting procedures to solve arithmetic problems did not. In all, we identified specific beginning of schooling numerical knowledge that contributes to individual differences in adolescents’ functional numeracy and demonstrated that performance on mathematical achievement tests underestimates the importance of this early knowledge.

## Introduction

A substantial number of adults have not mastered the mathematics expected of an eighth grader (22% in the U.S.) [1], making them functionally innumerate. They are not qualified for many jobs in today’s economy and have difficulty with now routine quantitative tasks [2]. Measures used in these economic studies typically include word problems that require whole number arithmetic, fractions, simple algebra, and measurement, with performance on these tests predicting employability and wages in adulthood, controlling for other factors [2]–[6]. Although items on numeracy measures overlap those on mathematics achievement tests, they are not entirely the same. Achievement tests include items that cover a broad range of mathematical content, whereas the functional numeracy measures provide a more focused assessment of mathematical competencies that influence economic opportunity and other real-world outcomes [7].

Early identification and remediation of knowledge deficits that predict long-term risk of innumeracy thus have the potential to yield substantial social and personal benefits [7]. Previous studies revealed that some aspects of young children’s basic knowledge of counting, numbers, and simple arithmetic predicts later mathematics achievement; specifically, skill at judging the relative magnitudes of Arabic numerals, the sophistication of the approaches they use to solve arithmetic problems, and an understanding of the mathematical number line [8]–[13]. Other studies suggest that sensitivity to the magnitudes of collections of objects may also contribute to mathematics achievement [14]. None of these studies provided an assessment of the relation between these basic quantitative competencies and later performance on functional numeracy measures. Moreover, studies showing that mathematics achievement in kindergarten predicts mathematics achievement throughout schooling [15] did not included measures that allowed for identification of the specific basic quantitative competencies that are critical for mathematics learning and those that are less central.

As part of a kindergarten to ninth grade longitudinal study of children’s mathematical development, in seventh grade we administered tests that are similar [2] or nearly identical [3] to the functional numeracy tests used in labor economic studies. Seventh graders of course are not ready for the workforce, but this assessment gauges their progress toward this critical end. The goal was to identify the key beginning of schooling basic quantitative competencies that contribute to seventh grade performance on these economically-relevant numeracy tests, while controlling for intelligence, working memory, in-class attentive behavior, and demographic factors that are predictive of mathematics learning and achievement [16], [17].

## Materials and Methods

### Ethics Statement

The study was reviewed and approved by the Institutional Review Board of the University of Missouri. Written consent was obtained from all parents, and all participants provided verbal assent for all assessments.

### Participants

The data are from a prospective longitudinal study of children’s mathematical development and risk of learning disability [18]. All kindergarten children from 12 elementary schools that serve families from a wide range of socioeconomic backgrounds were invited to participate. Parental consent and child assent were received for 37% (N = 311) of these children and 288 of them completed all or nearly all of the first year measures and 1 other child completed a subset of the measures (see [19]). The current analyses are based on 180 children (96 girls) who began the study in kindergarten and completed the functional numeracy testing in seventh grade. Across the seven years of data analyzed here, 4.7% of the observations were missing. Missing observations were estimated (maximum likelihood estimates with 5 imputations) using the multiple imputations program of SAS [20].

At the end of first grade, the intelligence of the sample was average (M = 102, SD = 15), based on the Wechsler Abbreviated Scale of Intelligence (WASI) [21]. At the end of kindergarten, the mathematics achievement of the sample was average (M = 102, SD = 13), but their reading achievement was high average (M = 112, SD = 14). At the end of seventh grade, mean mathematics (M = 95, SD = 19) and reading (M = 101, SD = 12) achievement were average [22].

The intelligence of the 109 children who did not participate in the seventh grade assessment was average (M = 94, SD = 15), but lower than that of the final sample (p<.0001). Their kindergarten mathematics achievement was average (M = 99, SD = 14) but slightly (d = .22) lower than that of the final sample (p = .01). Their reading achievement was high average (M = 110, SD = 16) and did not differ from that of the final sample (p = .16). The group differences, favoring the final sample, in intelligence and kindergarten mathematics achievement suggest that the results obtained in these analyses may be an underestimate of the actual relation between beginning of first grade early quantitative competencies assessed by mathematical cognition tasks (below) and seventh grade functional numeracy.

The mean age at the time of the first grade mathematical cognition assessment was 6.8 years (SD = 4 months) and 13.0 years (SD = 4 months) at the time of the seventh grade numeracy assessment. The racial composition was white (77%), Asian (5%), black (5%), and mixed race (8%), with the parents of the remaining children identifying them as Native American, Pacific Islander, or unknown. Across racial categories, 4% of the sample identified as ethnically Hispanic. Thirty-four percent of the children attending the schools from which the sample was drawn were eligible for free or reduced price lunches.

### Standardized Measures

#### Intelligence

The tests were the Colored Progressive Matrixes [23] (M = 102, SD = 14), and the Vocabulary and Matrix Reasoning subtests of the WASI (M = 102, SD = 15). The score used in the analyses was the mean of these two tests (M = 102, SD = 13, α = .76).

#### Achievement

Mathematics and reading achievement were assessed using the Numerical Operations and Word Reading subtests from the Wechsler Individual Achievement Test-II-Abbreviated [22].

### Mathematical Cognition Predictors

#### Addition strategy choices

Fourteen simple addition problems and six more complex problems were horizontally presented, one at a time, on flash cards in first grade and on the screen of a laptop computer thereafter. The simple problems consisted of the integers 2 through 9, with the constraint that the same two integers (e.g., 2+2) were never used in the same problem; ½ of the problems summed to 10 or less and the smaller valued addend appeared in the first position for ½ of the problems. The complex items were 16+7, 3+18, 9+15, 17+4, 6+19, and 14+8.

The child was asked to solve each problem (without pencil and paper) as quickly as possible without making too many mistakes. It was emphasized that the child could use whatever strategy was easiest to get the answer, and to speak the answer; from second grade forward, the answer was spoken into a voice activated microphone that recorded reaction time (RT) from problem onset. After solving each problem the child was asked to describe how they got the answer. Based on the child’s description and the experimenter’s observations, the trial was classified based on problem solving strategy. The four most common strategies were counting fingers, verbal counting, retrieval (quickly stating an answer and describing they “just remembered”), and decomposition (describing that they solved the problem by decomposing one addend and successively adding these smaller sets to the other addend; e.g., 17+8 = 17+3+5). Counting trials were further classified as min (stating the larger valued addend and counting the smaller one), sum (counting both addends starting from one), or max (stating the value of the smaller addend and then counting the larger one). The combination of experimenter observation and child reports immediately after each problem is solved has proven to be a useful measure of children’s strategy choices [24], [25]. The validity of this information is supported by previous studies that have demonstrated RT patterns vary systematically across strategies; finger-counting trials have the longest RTs, followed respectively by verbal counting, decomposition, and direct retrieval [25], [26].

The variables used here were the frequency with which min counting was correctly used to solve the simple problems and the more complex problems. The frequency of correctly retrieving the answers was also used for simple problems, and the frequency with which decomposition was correctly used for complex problems (Table 1).

**Table 1:** School Entry Mathematical Cognition Variables.

Variable	Factor	Operationalization
Simple addition counting	Counting Competence	Frequency and accuracy of use of mature procedure
Complex addition counting	Counting Competence	Frequency and accuracy of use of mature procedure
Simple addition retrieval	Number System Knowledge	Correct retrieval of answers to number combinations
Complex addition decomposition	Number System Knowledge	Frequency of correct use of decomposition
Number line accuracy	Number System Knowledge	Accuracy in placement of numerals on a number line
Number sets fluency	Number System Knowledge	Signal detection measure based on hits and misses

#### Number sets

**Figure 1:**
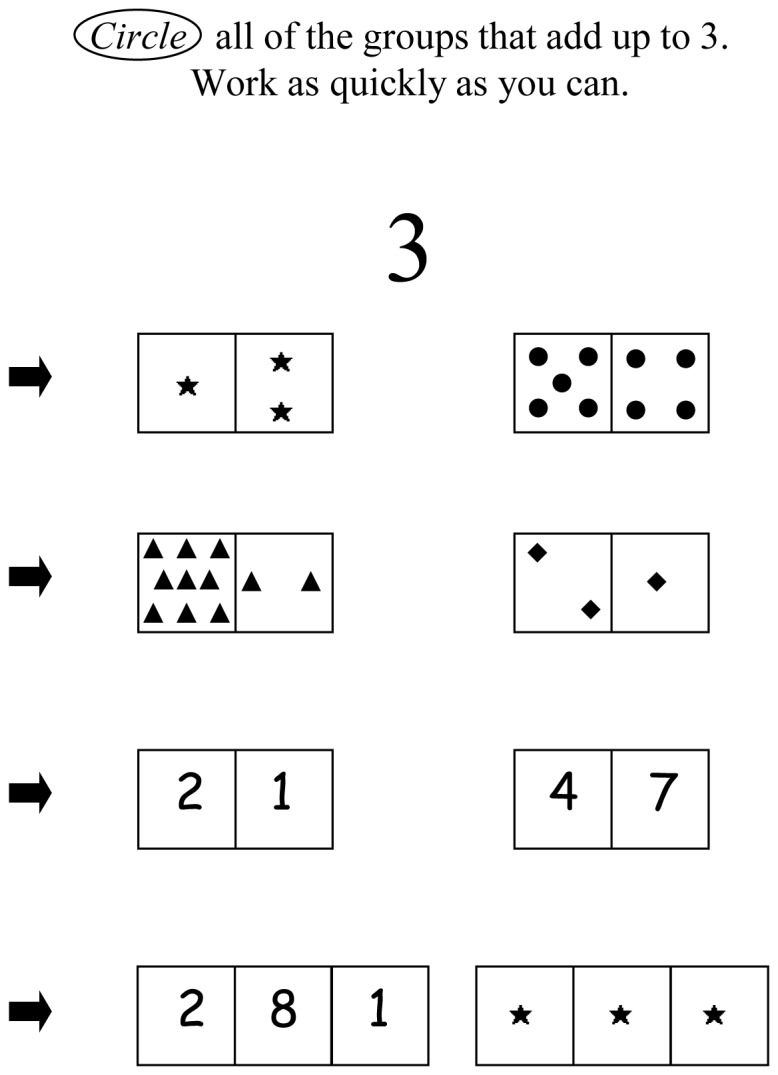
Practice items from the number sets measure. The task is to circle rectangles that contain collections of objects, Arabic numerals, or a combination that match a target number. For the actual task, children had 120 sec to identify which of 72 items matched a target of 5, and 180 sec for a target of 9.

Two types of stimuli were used: objects (e.g., stars) in a 1/2′′ square and an Arabic numeral (18 pt font) in a 1/2′′ square. Stimuli are joined in domino-like rectangles with different combinations of objects and numerals (Figure 1). These dominos are presented in lines of 5 across a page. The last two lines of the page show three 3-square dominos. Target numbers (5 or 9) are shown in large font at the top the page. On each of two pages for each target number, 18 items match the target; 12 are larger than the target; 6 are smaller than the target; and 6 contain 0 or an empty square.

The tester began by explaining two items that matched a target sum of 4; then, used the target sum of 3 for practice. The measure was then administered. The child was told to move across each line of the page from left to right without skipping any; to “circle any groups that can be put together to make the top number, 5 (9)”; and to “work as fast as you can without making many mistakes.” The child had 60 sec per page for the target 5; 90 sec per page for the target 9. Time limits were chosen to avoid ceiling effects and to assess fluent recognition and manipulation of quantities associated with collections of objects and Arabic numerals. Performance is consistent across target number and item content (e.g., whether the rectangle included Arabic numerals or objects) and thus these were combined to create an overall frequency of hits (α = .88), correct rejections (α = .85), misses (α = .70), and false alarms (α = .90) [27]. The variable used here was based on the signal detection d-prime measure; specifically, (standardized hits – standardized false alarms) [28].

After first grade, some of the children completed all items in less than the maximum times (120 and 180 sec for targets of 5 and 9, respectively) and thus their scores were adjusted upwards; specifically, (hits – false alarms)×(maximum RT/actual RT). The adjustment enabled us to maintain the sensitivity of the test, despite faster processing times across grades.

#### Number line estimation

A series of twenty-four 25 cm number lines containing a blank line with two endpoints (0 and 100) was presented, one at a time, to the child with a target number (e.g., 45) in a large font printed above the line. The child’s task was to mark the line where the target number (using pencil and paper in first grade and a laptop and mouse thereafter) should lie [29]. We used absolute accuracy in these analyses, because this is correlated with mathematics achievement [10], [29], [30]. Accuracy is defined as the absolute difference between the child’s placement and the correct position of the number (e.g., for the number 45, placements of 35 and 55 produce difference scores of 10). The overall score is the mean of these differences across the 24 trials.

The mechanisms that support children’s learning of the mathematical number line are debated [31]–[33]. Whatever the mechanisms, the key for academic mathematics is the insight that the distance between two consecutive whole numbers is the same, regardless of position on the line, that is, the line is linear. The extent to which children’s placements respect this linearity will be reflected in their absolute error on this task.

#### Factor analysis

The six mathematical cognition variables listed in Table 1 were submitted to a principal components factor analysis, with promax rotation [20], [34]. Two factors yielded Eigenvalues >1.0 (i.e., values of 2.6, 1.8) and in combination explained 73% of the covariation among the variables; the factors were not correlated (r = .12, p = .1112). The first factor, Counting Competence, was defined by the two min counting variables and the second by the four remaining variables (Table S1 in File S1). The number sets fluency variable loaded equally well on both factors. This may reflect the use of counting the collections of objects to solve some items and use of an understanding of magnitude to solve others. Because the number sets variable was strongly correlated with the number line variable (r = −.64, p = .0001; the correlation is negative because smaller errors on the number line task indicates better performance) and given the clear importance of the counting variables for defining the Counting Competence factor, the number sets variable was included as part of the Number System Knowledge factor. Composites were created by taking the standardized (M = 0, SD = 1) mean of the associated variables; α = .84 and.73 for the Counting Competence and Number system knowledge variables, respectively.

### Working Memory Predictors

The Working Memory Test Battery for Children (WMTB-C) [35] consists of nine subtests assessing the three core components of working memory (see Method and Materials in SI). The mean of the total scores for the corresponding subtests were used for the central executive (α = .75,.69 for first and fifth grade, respectively), phonological loop (α = .80,.78), and visuospatial sketch pad (α = .58,.60).

### In-Class Attentive Behavior Predictor

The Strength and Weaknesses of ADHD–symptoms and normal-behavior (SWAN) was used as the measure of in-class attentive behavior [36]. The items assess attentional deficits and hyperactivity but the scores are normally distributed, based on the behavior of a typical child in the classroom. The nine item (e.g., “Gives close attention to detail and avoids careless mistakes”) measure was distributed to the children’s second, third, and fourth grade teachers who were asked to rate the behavior of the child relative to other children of the same age on a 1 (far below) to 7 (far above) scale. At least one rating was available for 150 participants and multiple ratings were available for 120 to 125 participants. For the latter, the ratings were highly correlated across teachers, rs = .71 to.75 (ps<.0001), and thus we used the mean of available ratings (α = .88); missing data for the remaining 30 participants were imputed.

### Control Variables

The six control variables were sex, race, first grade school site, beginning of first grade speed of Arabic numeral encoding and articulation, and raw kindergarten Numerical Operations and Word Reading scores. The race variable provided separate contrasts of White children with Black children, White children with Asian children, and White children with all remaining children. The estimates for the race contrasts need to be interpreted with caution, given the small sample size for some of the contrasts (see Control Variables in SI). Their inclusion is important nonetheless as a control variable.

### Functional Numeracy Outcomes

The measures were selected based on labor economic studies of employability, wages, and related outcomes in adulthood [2]–[6] (see Method and Materials in SI).

#### Arithmetical word problems

Competence in solving multi-step word problems was assessed using the first form (15 items) of the Arithmetic Aptitude Test from the Educational Testing Service (ETS) kit of factor-referenced tests [37]. The score was the number of items solved correctly minus a fraction of the number of items solved incorrectly to control for guessing. The test has acceptable reliability estimates for adolescents (α = .61–.79) [38].

#### Computational arithmetic

The first form of three tests from the ETS kit [37] were used: Addition, (e.g., 12+42+53), Subtraction and Multiplication (e.g., 83−57; 85×6), and Division (e.g., 728÷8). For each test, the score was the number of problems solved correctly in 2 min. Performance across tests was highly correlated (rs = .61 to.77, ps<.0001) and thus scores were summed to create a composite, Arithmetic Computations measure (α = .88).

#### Computational fractions

Based on Hecht [39], three tests were used; Addition (e.g., 2 ¼+¼), Multiplication (e.g., ¼×1/8), and Division (e.g., 1/3÷1/6). For each test, the score was the number of problems solved correctly in 1 min.

The mean score for the division test was 1.4 (SD = 2.5) problems solved correctly and the median was 0 (75% of the participants did not solve a single problem correctly), a pattern of very low performance that was also found by Siegler et al. [40] for a nationally representative sample of U.S. students. The mean score for multiplication was 2.4 (SD = 2.9) and the median was 1 (none of the students below the median solved any problems correctly). Based on the low variation for multiplication and division, and their low correlation with the addition scores (rs = .24,.35, respectively), these measures were dropped. The mean for addition was 6.4 (SD = 3.7) and the median was 6 (90% of the participants correctly solved at least one problem).

#### Fractions comparison test

The test is composed of 16 pairs of fractions and was developed based on children’s common problem solving errors or the strategies they use when solving fractions problems [39], [41]. For each pair the child is asked to circle the larger of the two fractions and is given 120 sec to complete the test. The pairs vary in terms of the relations among the numerators and denominators (four items for each type). In the first type the numerator is constant but the denominator differs (e.g., 2/4 2/5), which assesses children’s understanding of the inverse relation between the value of the denominator and the quantity represented by the fraction. The larger fraction will have the smaller denominator. In the second type numerators have a ratio of 1.5 and denominators a ratio between 1.1 and 1.25 (e.g., 3/10 2/12), making identification of the larger magnitude easier using numerators (larger value is correct), whereas focus on the denominators will result in errors (larger value is incorrect). The ratios were determined based on the Weber fraction for ease of magnitude discrimination for adolescents [42]. In the third type numerators and denominators are reversed (e.g., 5/6 6/5), which requires children to choose the fraction with the larger numerator and smaller denominator. The final type involves skill at using ½ as an anchor for estimating fraction values (e.g., 20/40 8/9). The foils are always close to one but contain smaller numerals than the ½ fraction. A child who understands fractions should be able to quickly determine that one fraction equals ½ and the other fraction is close to one and thus choose the latter. A child who focuses on absolute magnitude of the numbers that compose the fractions will choose the ½ fractions and thus commit errors.

Answers were scored as hits (coded 1) or misses (coded −1). Hits were significantly correlated across the four problem types (rs = .39 to.74, ps<.0001) and thus summed to create a total hits variable (α = .81). Misses were also significantly correlated (rs = .36 to.74, p<.0001) and summed (α = .79). The fractions comparison score was hits minus misses. The validity of the measure was demonstrated by showing that scores predict one year gains in mathematics achievement, controlling for previous mathematics achievement, intelligence, and working memory [43].

#### Factor analysis

The four word problem, computational arithmetic, and fractions measures were submitted to a principal components factor analysis, which yielded a single factor (Eigenvalue = 2.6) that explained 66% of the covariation among the variables (factor loadings >.76). A Functional Numeracy composite was created by taking the standardized mean of the four variables (*α* = .83).

### Procedure

The CPM and WASI were administered in the spring of kindergarten and first grade, respectively, and the achievement tests were administered every spring beginning in kindergarten. The mathematical cognition tasks were administered once a year, beginning in the fall of first grade. The WMTC-B was administered in first (M = 84 months, SD = 6) and fifth (M = 128 months, SD = 5) grades (Table S2 in File S1). The numeracy tests were generally administered to groups of about 5 to 20 between the fall and spring seventh grade assessments.

## Results

### First Grade Number System Knowledge Predicts Seventh Grade Functional Numeracy

Adolescents’ scores on the functional numeracy measure were significantly correlated with their beginning of first grade counting competence (r = .31, p<.0001) and number system knowledge (r = 0.69, p<.0001) scores (Table S3 in File S1). Regression analyses indicated that scores on the number system knowledge variable remained predictive of functional numeracy (ß = 0.287, p<.0001), with simultaneous estimation of the control, intelligence, working memory, and in-class attentive behavior variables (Table 2). In contrast, counting competence did not predict functional numeracy (p = .40), when all other variables were included in the regression equation.

**Table 2:** Ordinary Least Squares Estimates (± standard errors) for Prediction of Adolescent Functional Numeracy.

	Prediction of Functional Numeracy		
Effect	Estimates	t	p
Intercept	0.248±0.115	2.15	0.0332
**Control Variables**			
Girls contrasted with boys	−0.096±0.118	−0.81	0.4183
Mixed race contrasted with White	−0.096±0.119	−0.81	0.4212
Black contrasted with White	0.020±0.216	0.09	0.9280
Asian contrasted with White	0.508±0.190	2.67	0.0084
Kindergarten mathematics achievement	0.108±0.056	1.94	0.0540
Kindergarten reading achievement	0.002±0.063	0.03	0.9755
Number processing speed	−0.003±0.051	−0.06	0.9526
**Cognitive and In-Class Predictors**			
Intelligence	0.105±0.065	1.62	0.1081
First grade phonological loop	−0.047±0.071	−0.66	0.5086
First grade visuospatial sketch pad	−0.077±0.055	−1.40	0.1645
First grade central executive	0.023±0.064	0.37	0.7097
Fifth grade phonological loop	0.000±0.060	0.01	0.9936
Fifth grade visuospatial sketch pad	0.043±0.054	0.80	0.4272
Fifth grade central executive	0.130±0.060	2.18	0.0307
In-class attentive behavior	0.167±0.057	2.93	0.0039
**First Grade Mathematical Cognition Measures**			
Counting Competence	0.044±0.051	0.85	0.3984
Number System Knowledge	0.287±0.070	4.00	0.0001

R^2^ = .68, F_28,151_ = 11.63, p<.0001. The school site contrast is not shown and was not significant (p = .36).

The same analyses were conducted for each of the four tests that composed the functional numeracy composite (Table S4 in File S1). The counting competence variable was never significant (ßs = −0.091 to 0.124, ps>.12) and the number system knowledge variable was always significant (ßs = 0.246 to 0.348, ps<.014).

### Achievement Tests Underestimate Numeracy Deficits

Seventh grade mathematics achievement and functional numeracy scores were significantly correlated, r = .79, p<.0001, but less so once fifth grade working memory (the assessment closest to seventh grade), intelligence, and in-class attentive behavior were controlled, pr = .50, p<.0001. At noted earlier, the functional numeracy measures have been shown to be predictive of important life outcomes in adults [2]. However, if achievement tests predict outcomes in adulthood as well as functional numeracy tests or if the number system knowledge or counting competence measures used in this study (or measures of any other early core mathematics competence) predict later achievement as strongly as they predict later numeracy, then the two types of measures are interchangeable, that is, use of functional numeracy measures provides no added utility beyond that provided by standard mathematics achievement tests.

Number system knowledge remained predictive of functional numeracy, after controlling for seventh grade mathematics achievement (ß = 0.195, p = .0014; Table S5 in File S1), but did not predict seventh grade mathematics achievement after controlling for functional numeracy (ß = 0.014, p = .8760; Table S6 in File S1).

Logistic regression revealed a 1 *SD* decrease in number system knowledge scores in first grade resulted in a 4.14 fold increase in the odds of scoring in the bottom quartile on the functional numeracy measure in seventh grade [χ^2^(1) = 3.92, p = .0479, 95% confidence interval, 1.01–16.88], controlling for all variables in Table 2 and seventh grade mathematics achievement. In contrast, poor number system knowledge scores did not predict the odds of being in the bottom quartile of seventh grade mathematics achievement, controlling for all variables in Table 2 and functional numeracy scores [odds = 1.28, χ^2^(1) = 0.19, p = .6664, 95% confidence interval, 0.42–3.90].

### Growth in Number System Knowledge and Functional Numeracy

The analyses thus far indicate that children who begin first grade with low number system knowledge are at heightened risk for low functional numeracy scores in seventh grade. As a follow up, we sought to determine whether first-to-fifth grade growth in number system knowledge is also related to functional numeracy in seventh grade.

The measures that defined the Number System Knowledge factor were administered in first through fifth grade, inclusive. A principle components factor analysis, with promax rotation confirmed that the four variables defined the same Number System Knowledge factor identified for first grade in second to fifth grade, inclusive (Eigenvalues >1.76, factor loaders>|.54|).

To make each measure comparable to the others and across grades, the associated scores were defined as the percentage of maximum possible performance; specifically, for simple addition (number of problems correctly retrieved/14), for complex addition (number of problems correctly solved with decomposition/6), for Number Sets (RT adjusted d-prime score/maximum score achieved in fifth grade across all children), and number line [1– (mean error/50)]. Fifty was chosen for the latter, because random placements would, on average, result in mean errors of 50 on the 0-to-100 number line. A child making random placements would thus have a score of 1–1, or 0 percent. The most accurate child in our study had a mean error of 1.75 in fifth grade, resulting in a score of 0.965.

**Figure 2:**
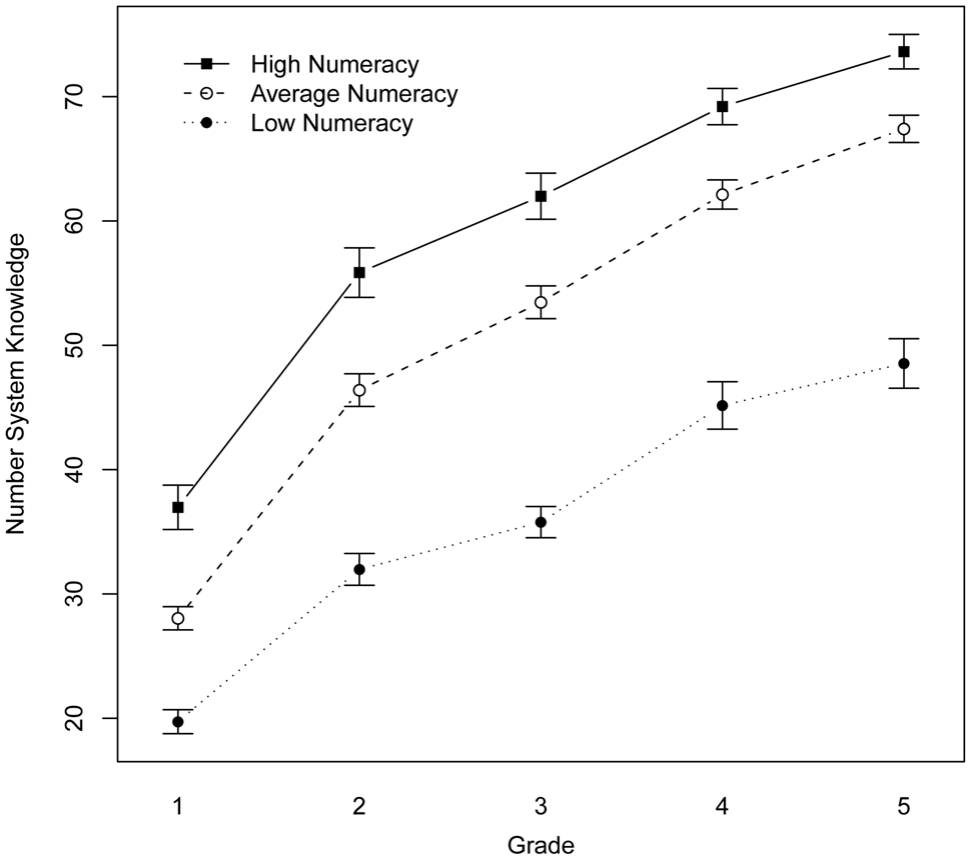
Growth in Number Systems Knowledge across grades for the bottom (Low), two middle (Average), and top (High) quartiles on the seventh grade numeracy measure. The score is the percentage of the maximum possible score across the four tasks that composed the Number System Knowledge factor.

Children who scored in the bottom quartile on the functional numeracy measure had a lower number system knowledge start point and slower first to fifth grade growth than children in the top and middle quartiles (ps<.0803; Figure 2, see Growth in Number System Knowledge in SI). The two latter groups differed for start point (p = .0225), but not growth (ps>.5275). The slow growth of the low group, however, was due entirely to group differences in rate of improvement from first to second grade. From second to fifth grade, the rate of improvement in number system knowledge did not differ comparing any of the groups (ps>.3526).

## Discussion

The results provide three key insights into children’s mathematical development. The first is that some aspects of their school entry quantitative knowledge, as measured by the mathematical cognition tasks, contribute to long-term functional numeracy, controlling other factors that affect learning, whereas other aspects of their knowledge do not. Of particular importance were the competencies common to the measures that defined the Number System Knowledge factor. All of these measures require explicit processing of Arabic numerals and operating on them in ways consistent with the logical, systematic relations among numerals. At school entry, this emerging knowledge of the number system includes an understanding of the relative magnitude of numerals, their ordering, and the ability to combine and decompose them into smaller and larger numerals and to use this knowledge to solve arithmetic problems. Whether or not this explicit number system knowledge is dependent on a potentially inherent sense of magnitude for its initial development [14] or develops independently [8], [44], [45] remains to be determined.

At the same time, children’s skill at using counting procedures to solve addition problems at the beginning of first grade was not predictive of their later functional numeracy scores, holding other factors constant. One potential reason for this is because children who begin school behind their peers in the use of these counting procedures tend to catch up with other children within one or two years [26]. It is very likely that competence at using more complex mathematical procedures, as in borrowing or carrying to solve multi-column arithmetic problems, contributes to functional numeracy. Indeed, functional numeracy measures include problems that require use of these more complex procedures.

The second key finding is the previously noted relation between mathematics achievement in kindergarten and mathematics achievement throughout schooling [15] may underestimate the long-term consequences of poor school entry quantitative knowledge. The functional numeracy measures have been validated through their ability to predict economic opportunity and day-to-day competence with routine quantitative tasks [5], [7], and school entry number system knowledge predicts functional numeracy, even with the control of same-grade mathematics achievement. Critically, number system knowledge does not predict mathematics achievement, once functional numeracy is controlled. In short, the functional numeracy assessment appears to capture individual differences in adolescents’ developing economically-relevant competencies above and beyond those captured by standard mathematics achievement tests.

The third key finding is that growth in number system knowledge is less important for predicting functional numeracy than is school entry number system knowledge. Children scoring in the bottom quartile on the numeracy measure in seventh grade started school behind their peers in number system knowledge and showed less rapid growth from first to second grade, but typical growth thereafter. Future studies are needed to determine how this early number system knowledge influences the learning of more complex aspects of the number system (e.g., the base-10 organization), and how this influences emerging functional numeracy. For now, the implication is that interventions to improve children’s early understanding of the relations among numerals need to be implemented before the start of schooling or in first grade, and fortunately such interventions are being developed [46], [47].

## Supporting Information

File S1 This file contains: Method and Materials–provides detailed description of the working memory and functional numeracy measures; Control Variables–provides detailed description of the control variables; Table S1–Standardized Factor Loadings for the Mathematical Cognition Measures in First Grade; Table S2–Overall Design of the Missouri Study; Table S3–Means and Correlations Among Variables. All variables were standardized (M = 0, SD = 1) and analyzed in PROC GLM [20]. The data were also analyzed in PROC MIXED with maximum likelihood and restricted maximum likelihood estimation of parameters, with the same results; Table S4–Ordinary Least Squares Estimates (± standard errors) for Prediction of Individual Measures that Composed the Functional Numeracy Composite. The full model R^2^s = .55,.59,.51, and.48 (ps<.0001) for the word problems, computational arithmetic, computational fractions, and fractions concepts scores, respectively. The school site contrasts are not shown and were not significant in any equation (ps>0.08); Table S5–Ordinary Least Squares Estimates (± standard errors) for Prediction of Adolescent Functional Numeracy Controlling for Seventh Grade Mathematics Achievement. R^2^ = .78, F_29,150_ = 18.18, p<0001. The school site contrast is not shown and was not significant (p = .43); Table S6–Ordinary Least Squares Estimates (± standard errors) for Prediction of Seventh Grade Mathematics Achievement Controlling for Functional Numeracy. R^2^ = .70, F_29,150_ = 12.18, p<.0001. The school site contrast is not shown and was not significant (p = .69); Growth in Number System Knowledge–provides detailed analyses on the creation of the across-grade Number System Knowledge variable.
(DOCX)
