# Supplementary material for: Adolescents’ Functional Numeracy Is Predicted by Their School Entry Number System Knowledge
Source: PLoS One. 2013 Jan 30;8(1):e54651. doi: 10.1371/journal.pone.0054651 (PMC3559782; doi:10.1371/journal.pone.0054651)
Supplement: File S1 — This file contains: Method and Materials–provides detailed description of the working memory and functional numeracy measures; Control Variables–provides detailed description of the control variables; Table S1–Standardized Factor Loadings for the Mathematical Cognition Measures in First Grade; Table S2–Overall Design of the Missouri Study; Table S3–Means and Correlations Among Variables. All variables were standardized (M = 0, SD = 1) and analyzed in PROC GLM [20]. The data were also analyzed in PROC MIXED with maximum likelihood and restricted maximum likelihood estimation of parameters, with the same results; Table S4–Ordinary Least Squares Estimates (± standard errors) for Prediction of Individual Measures that Composed the Functional Numeracy Composite. The full model R2s = .55,.59,.51, and.48 (ps<.0001) for the word problems, computational arithmetic, computational fractions, and fractions concepts scores, respectively. The school site contrasts are not shown and were not significant in any equation (ps>0.08); Table S5–Ordinary Least Squares Estimates (± standard errors) for Prediction of Adolescent Functional Numeracy Controlling for Seventh Grade Mathematics Achievement. R2 = .78, F29,150 = 18.18, p<0001. The school site contrast is not shown and was not significant (p = .43); Table S6–Ordinary Least Squares Estimates (± standard errors) for Prediction of Seventh Grade Mathematics Achievement Controlling for Functional Numeracy. R2 = .70, F29,150 = 12.18, p<.0001. The school site contrast is not shown and was not significant (p = .69); Growth in Number System Knowledge–provides detailed analyses on the creation of the across-grade Number System Knowledge variable. (DOCX) [file pone.0054651.s001.docx]

**Supplementary Material** for D. C. Geary, M. K. Hoard, L. Nugent, & D. H. Bailey, “Adolescents’ Functional Numeracy is Predicted by Their School Entry Number System Knowledge”

**METHOD And MATERIALS**

Working Memory

The Working Memory Test Battery for Children (WMTB-C) [1] consists of nine subtests that assess the central executive, phonological loop, and visuospatial sketchpad components of working memory [2]. All of the subtests have six items at each span level. Across subtests, the span levels range from one to six to one to nine. Passing four items at one level moves the child to the next. At each span level, the number of items (e.g., words) to be remembered is increased by one. Failing three items at one span level terminates the subtest. Working memory scores for the central executive (α = .75, .69 for first and fifth grade, respectively), phonological loop (α = .80, .78), and visuospatial sketch pad (α = .58, .60) are the mean of the total scores for the corresponding subtests.

*Central executive*. The central executive is assessed using three dual-task subtests. Listening Recall requires the child to determine if a sentence is true or false, and then recall the last word in a series of sentences. Counting Recall requires the child to count a set of 4, 5, 6, or 7 dots on a card, and then to recall the number of counted dots at the end of a series of cards. Backward Digit Recall is a standard format backward digit span.

*Phonological loop.* Digit Recall, Word List Recall, and Nonword List Recall are standard span tasks with differing content stimuli; the child’s task is to repeat words spoken by the experimenter in the same order as presented by the experimenter. In the Word List Matching task, a series of words, beginning with two words and adding one word at each successive level, is presented to the child. The same words, but possibly in a different order, are then presented again, and the child’s task is to determine if the second list is in the same or different order than the first list.

*Visuospatial sketch pad*. Block Recall is another span task, but the stimuli consist of a board with nine raised blocks in what appears to the child as a “random” arrangement. The blocks have numbers on one side that can only be seen from the experimenter’s perspective. The experimenter taps a block (or series of blocks), and the child’s task is to duplicate the tapping in the same order as presented by the experimenter. In the Mazes Memory task, the child is presented a maze with more than one solution in a response booklet, and a picture of an identical maze with a path drawn for one solution. The latter picture is removed and the child’s task is to duplicate in the path in the response booklet. At each level, the mazes get larger by one wall.

Functional Numeracy Measures

Functional numeracy measures typically include word problems that require whole number arithmetic, fractions, simple algebra, and measurement, and performance on these tests predicts employability, wages, on the job productivity, as well as competence in dealing with day-to-day quantitative tasks, in adults up to at least 37 years of age, controlling for sex, race, intelligence, and reading ability [3-6].

To illustrate the assessments conducted in this type of research, consider the measures used in Berlin and Sum’s [7] study of the relation between basic academic competencies and wage stagnation (controlling for inflation) between 1973 and 1984 for a nationally (U.S.) representative sample of 12,693 15 to 23 year olds. Academic competencies were assessed using the Armed Forces Qualifying Test (AFQT) for 11,914 of these individuals. The AFQT included (it has since been revised) ten subtests, four of which were used to derive the AFQT scores. These were word knowledge, reading, arithmetic reasoning, and numerical operations. The arithmetic reasoning subtest included 30 multi-step word problems and the numerical operations subtest is a timed 50 item measure of computational addition, subtraction, multiplication, and division. Kirsch and Jungeblut [8] assessed the basic skills of 3,600 young adults in the U.S. The tests assessed their ability to negotiate many routine day-to-day activities, such as determining a 10% tip for a meal bill (an item passed by 23% of the sample). Their “quantitative literacy” measure included simple whole number and fractional arithmetic problems embedded in single-step and multi-step word problems. The functional numeracy tests used in the United Kingdom are essentially the same [9].

In sum, the functional numeracy tests administered to our seventh graders are highly similar to those used in these economic studies. Performance on these measures gauges their progress toward being able to function well in a modern economy, including their ability to cope with many day-to-day routine activities, as well as their employment and wage prospects.

**CONTROL VARIABLES**

The six control variables were sex, race, school, beginning of first grade speed of Arabic numeral encoding and articulation, and raw kindergarten Numerical Operations and Word Reading scores. The race variable provided separate contrasts of White children with Black children, White children with Asian children, and White children with all remaining children. The results for the race contrasts need to be interpreted with caution, given the smaller sample sizes for the non-white groups than the white group. Their inclusion is important nonetheless as a control for potential race differences on the functional numeracy measure.

The school variable included the 12 schools for the sample when they were in first grade, and contrasted each of 11 schools with the school with the largest initial sample. The Numerical Operations score controlled for basic mathematics skills before entry into first grade. The Numerical Operations scores from seventh grade were also used as controls in some analyses.

Speed of encoding Arabic numerals and articulating number words are basic number processes that may affect children’s early skill at solving mathematics problems, and controlling for them allowed us to rule out these processes as potential contributors to the seventh grade outcomes [10]. Speed of encoding and articulation was assessed using a standard procedure, that is, the rapid automatized naming (RAN) task [11,12]. The child is presented with 5 numbers to first determine if the child can read the stimuli correctly. After these practice items, the child is presented with a 5 X 10 matrix of incidences of these numbers, and is asked to name them as quickly as possible without making any mistakes. RT is measured via a stopwatch.

**Table S1 Standardized Factor Loadings for the Mathematical Cognition Measures in First Grade. The negative loadings for number line accuracy indicate better performance (i.e., smaller error).**

| Variable | Counting Competence | Number System Knowledge |
| --- | --- | --- |
| Simple addition counting | 0.904 | -0.185 |
| Complex addition counting | 0.904 | -0.030 |
| Simple addition retrieval | -0.052 | 0.798 |
| Complex addition decomposition | -0.337 | 0.814 |
| Number line accuracy | -0.449 | -0.646 |
| Number sets fluency | 0.568 | 0.569 |

**Table S2 Overall Design of the Missouri Study. X indicates an assessments occurred and yellow indicates assessments used in the current analyses**

| **Grade** | **K** | **1** | **2** | **3** | **4** | **5** | **6** | **7** |
| --- | --- | --- | --- | --- | --- | --- | --- | --- |
| **Mathematics, Reading Achievement** | **X** | **X** | **X** | **X** | **X** | **X** | **X** | **X** |
| **Intelligence** | **X** | **X** |  |  |  |  |  |  |
| **Mathematical Cognition** | | **X** | **X** | **X** | **X** | **X** | **X** | **X** |
| **Speed of Arabic Numeral Encoding and Articulation** | | **X** | **X** | **X** | **X** | **X** | **X** | **X** |
| **Work Memory** | | **X** |  |  |  | **X** |  |  |
| **In-Class Attentive Behavior** | | | **X** | **X** | **X** |  |  |  |
| **Functional Numeracy** | | | | | | |  | **X** |

The Colored Progressed Matrixes [13] and the Wechsler Abbreviated Scale of Intelligence [14] were administered in the spring of kindergarten and first grade, respectively, and the achievement tests were administered in the spring of each grade. The mathematical cognition and RAN tasks used here were administered in the fall of each grade; the one exception was the number line task, which was moved to the spring assessments beginning in second grade (due to testing time constraints in the fall). The majority of children were tested in a quiet location at their school site, and occasionally on the university campus or in a mobile testing van. Testing in the van occurred for children who had moved out of the school district or to a non-participating school and for administration of the Working Memory Test Battery —Children [1] (e.g., on the weekend or after school). The WMTC-B was administered in first (mean age = 84 months, *SD* = 6) and fifth (*M* = 128 months, *SD* = 5) grades. The functional numeracy tests were generally administered to groups of about 5 to 20 in the late fall and early spring of seventh grade at a mean age of 156 (*SD* = 4) months; the one exception was the fractions comparison test which was administered in the spring of sixth grade. The arithmetical and numerical cognition and achievement assessments required between 20 and 40 min¸ the WMTB-C about 60 min per assessment, and the functional numeracy assessment about 45 min.

**Table S3 Means and Correlations Among Variables.** All variables were standardized (*M* = 0, *SD* = 1) and analyzed in PROC GLM [15]. The data were also analyzed in PROC MIXED with maximum likelihood and restricted maximum likelihood estimation of parameters, with the same results.

| Variable | *M* | *SD* | 1 | 2 | 3 | 4 | 5 | 6 | 7 | 8 | 9 | 10 | 11 | 12 | 13 | 14 | 15 | 16 | 17 | 18 | 19 | 20 | 21 |
| --- | --- | --- | --- | --- | --- | --- | --- | --- | --- | --- | --- | --- | --- | --- | --- | --- | --- | --- | --- | --- | --- | --- | --- |
| 1: Word Problems | 2.8 | 3.2 | -- |  |  |  |  |  |  |  |  |  |  |  |  |  |  |  |  |  |  |  |  |
| 2: Comp: Arithmetic | 25.4 | 12.2 | 64 | -- |  |  |  |  |  |  |  |  |  |  |  |  |  |  |  |  |  |  |  |
| 3: Fractions: Addition | 6.4 | 3.7 | 56 | 58 | -- |  |  |  |  |  |  |  |  |  |  |  |  |  |  |  |  |  |  |
| 4: Fractions: Comparison | 22.2 | 16.7 | 52 | 51 | 48 | -- |  |  |  |  |  |  |  |  |  |  |  |  |  |  |  |  |  |
| 5: PL: 1^st^ Grade | 78.8 | 14.3 | 31 | 37 | 25 | 38 | -- |  |  |  |  |  |  |  |  |  |  |  |  |  |  |  |  |
| 6: PL: 5^th^ Grade | 90.0 | 13.8 | 31 | 30 | 15 | 31 | 73 | -- |  |  |  |  |  |  |  |  |  |  |  |  |  |  |  |
| 7: VSSP: 1^st^ Grade | 28.1 | 7.1 | 27 | 41 | 27 | 27 | 45 | 25 | --- |  |  |  |  |  |  |  |  |  |  |  |  |  |  |
| 8: VSSP: 5^th^ Grade | 45.0 | 8.8 | 33 | 45 | 41 | 41 | 30 | 26 | 57 | -- |  |  |  |  |  |  |  |  |  |  |  |  |  |
| 9: CE: 1^st^ Grade | 33.1 | 9.4 | 39 | 50 | 32 | 42 | 64 | 49 | 57 | 47 | -- |  |  |  |  |  |  |  |  |  |  |  |  |
| 10: CE: 5^th^ Grade | 49.4 | 10.0 | 48 | 50 | 36 | 48 | 55 | 54 | 44 | 48 | 62 | -- |  |  |  |  |  |  |  |  |  |  |  |
| 11: Cognitive ability | 102 | 13 | 49 | 31 | 43 | 42 | 50 | 29 | 42 | 37 | 46 | 43 | -- |  |  |  |  |  |  |  |  |  |  |
| 12: In-class attention | 4.8 | 1.3 | 42 | 53 | 40 | 46 | 45 | 39 | 41 | 51 | 57 | 57 | 31 | -- |  |  |  |  |  |  |  |  |  |
| 13: Number Naming RT | 42 | 12 | -29 | -43 | -28 | -28 | -38 | -33 | -35 | -35 | -48 | -41 | -24 | -24 | -- |  |  |  |  |  |  |  |  |
| 14: Kindergarten math | 55 | 25 | 38 | 43 | 40 | 34 | 41 | 30 | 40 | 37 | 47 | 35 | 39 | 38 | -43 | --- |  |  |  |  |  |  |  |
| 15: Kindergarten reading | 72 | 21 | 36 | 43 | 34 | 35 | 55 | 46 | 36 | 31 | 57 | 52 | 50 | 46 | -59 | 58 | --- |  |  |  |  |  |  |
| 16: Simple add retrieval | 1.7 | 2.1 | 43 | 31 | 29 | 37 | 18 | 07 | 14 | 23 | 21 | 18 | 29 | 17 | -13 | 27 | 23 | --- |  |  |  |  |  |
| 17: Simple add counting | 6.0 | 4.1 | 07 | 21 | 28 | 21 | 34 | 25 | 21 | 20 | 30 | 21 | 27 | 23 | -31 | 28 | 36 | -13 | --- |  |  |  |  |
| 18: Com add decomp | 0.3 | 1.0 | 40 | 31 | 27 | 24 | 03 | 06 | 18 | 27 | 12 | 14 | 17 | 06 | -08 | 35 | 12 | 45 | -22 | --- |  |  |  |
| 19: Com add counting | 3.1 | 2.3 | 21 | 32 | 30 | 29 | 48 | 37 | 27 | 16 | 41 | 30 | 32 | 42 | -37 | 37 | 47 | 13 | 72 | -23 | --- |  |  |
| 20: Number line | 14 | 7.7 | -42 | -38 | -47 | -51 | -42 | -29 | -34 | -40 | -45 | -42 | -50 | -32 | 34 | -46 | -45 | -40 | -30 | -32 | -37 | --- |  |
| 21: Number sets | 30 | 13 | 56 | 55 | 59 | 55 | 45 | 28 | 44 | 49 | 54 | 51 | 59 | 45 | -41 | 58 | 50 | 42 | 39 | 34 | 48 | -64 | --- |

Note: Correlations were multiplied by 100; Correlations > |14|, *p*<.05. Variables in red composed the functional numeracy measure; First grade working memory measures are in green and fifth grade measures in blue; number system knowledge measures are in brown and counting competence in purple. RT = reaction time (sec); Fractions: Comparison = hits – misses adjusted for RT on the fractions comparison test (Max = 74); Comp = computational; PL = Phonological Loop; VSSP = Visuospatial Sketch Pad; CE = Central Executive; Simple add retrieval = number of problems (e.g., 4 + 6) correctly solved by direct retrieval of the answer (max = 14); Simple add counting = number of simple problems correctly solved with the min procedure (max = 14); Com add decomp = number of complex addition problems (e.g., 16 + 8) solved by decomposing the smaller addend into sets (e.g., 8 = 4 + 4) and then successively adding these to the larger addend (max = 6); Com add counting = number of complex problems correctly solved with the min procedure (max = 6); Number line = absolute error and thus smaller values indicate better performance; Number sets = hits – misses on the numbers sets test (max = 72).

**Table S4 Ordinary Least Squares Estimates for Prediction of Individual Measures that Composed the Functional Numeracy Composite.** The full model *R*^2^s = .55, .59, .51, and .48 (*p*s < .0001) for the word problems, computational arithmetic, computational fractions, and fractions concepts scores, respectively. The school site contrasts are not shown and were not significant in any equation (*p*s > 0.08).

|  | **Word Problems** | | | **Computational Arithmetic** | | | **Computational Fractions** | | | **Fractions Concepts** | | | |
| --- | --- | --- | --- | --- | --- | --- | --- | --- | --- | --- | --- | --- | --- |
| Effect | Estimates | *t* | *P* | Estimates | *t* | *P* | Estimates | *t* | *P* | Estimates | *t* | | *P* |
| Intercept | 0.178 | 1.06 | 0.293 | 0.174 | 1.09 | 0.279 | 0.351 | 1.98 | 0.049 | 0.287 | 1.58 | | 0.116 |
| **Control Variables** | | | | | | | | | | | | | |
| Girls contrasted with boys | -0.156 | -1.18 | 0.238 | 0.115 | 0.92 | 0.360 | -0.161 | -1.16 | 0.249 | -0.169 | -1.18 | 0.240 | |
| Mixed race contrasted with White | -0.220 | -1.27 | 0.206 | 0.199 | 1.20 | 0.230 | -0.061 | -0.33 | 0.740 | -0.303 | -1.62 | 0.107 | |
| Black contrasted with White | -0.100 | -0.32 | 0.752 | 0.381 | 1.27 | 0.207 | -0.290 | -0.87 | 0.384 | 0.087 | 0.25 | 0.800 | |
| Asian contrasted with White | 0.679 | 2.44 | 0.016 | 1.285 | 4.85 | .0.000 | 0.208 | 0.71 | 0.479 | -0.137 | -0.46 | 0.648 | |
| Kindergarten mathematics achievement | 0.121 | 1.48 | 0.140 | 0.137 | 1.77 | 0.079 | 0.166 | 1.94 | 0.055 | 0.009 | 0.10 | 0.918 | |
| Kindergarten reading achievement | 0.134 | 1.46 | 0.146 | -0.030 | -0.34 | 0.736 | 0.029 | 0.30 | 0.763 | -0.126 | -1.27 | 0.206 | |
| Number processing speed | 0.019 | 0.26 | 0.796 | -0.110 | -1.56 | 0.120 | 0.028 | 0.36 | 0.717 | 0.051 | 0.63 | 0.527 | |
| **Cognitive and In-Class Predictors** | | | | | | | | | | | | | |
| Cognitive ability | 0.208 | 2.19 | 0.030 | -0.088 | -0.98 | 0.331 | 0.161 | 1.61 | 0.109 | 0.139 | 1.36 | 0.177 | |
| 1^st^ grade phonological loop | -0.196 | -1.90 | 0.060 | -0.008 | -0.08 | 0.937 | -0.010 | -0.09 | 0.926 | 0.027 | 0.24 | 0.812 | |
| 1^st^ grade visuospatial sketch pad | -0.067 | -0.83 | 0.409 | -0.020 | -0.26 | 0.795 | -0.088 | -1.04 | 0.298 | -0.133 | -1.53 | 0.129 | |
| 1^st^ grade central executive | 0.000 | 0.00 | 1.000 | 0.156 | 1.76 | 0.080 | -0.110 | -1.12 | 0.263 | 0.049 | 0.48 | 0.629 | |
| 5^th^ grade phonological loop | 0.130 | 1.49 | 0.139 | -0.037 | -0.83 | 0.653 | -0.097 | -1.07 | 0.288 | 0.007 | 0.08 | 0.939 | |
| 5^th^ grade visuospatial sketch pad | -0.070 | -0.88 | 0.381 | 0.072 | 0.92 | 0.345 | 0.113 | 1.36 | 0.177 | 0.058 | 0.68 | 0.500 | |
| 5^th^ grade central executive | 0.160 | 1.83 | 0.069 | 0.087 | 1.05 | 0.296 | 0.075 | 0.82 | 0.414 | 0.198 | 2.11 | 0.037 | |
| In-class attentive behavior | 0.140 | 1.68 | 0.095 | 0.134 | 1.69 | 0.094 | 0.185 | 2.11 | 0.036 | 0.210 | 2.33 | 0.021 | |
| **First Grade Mathematical Cognition Measures** | | | | | | | | | | | | | |
| Counting Competence | -0.091 | -1.21 | 0.229 | 0.105 | 1.47 | 0.143 | 0.124 | 1.57 | 0.118 | 0.036 | 0.44 | 0.662 | |
| Number System Knowledge | 0.285 | 2.77 | 0.006 | 0.246 | 2.51 | 0.013 | 0.269 | 2.48 | 0.014 | 0.348 | 3.13 | 0.002 | |

The analyses reported in the main article for the composite functional numeracy variable were redone for each of the four variables that composed the composite.

**Table S5 Ordinary Least Squares Estimates (± standard errors) for Prediction of Adolescent Numeracy Controlling for Seventh Grade Mathematics Achievement.** *R*^2^ = .78, *F*_29,150_ = 18.18, *p*< .0001. The school site contrast is not shown and was not significant (*p* = .43).

|  | **Prediction of Functional Numeracy** | | |
| --- | --- | --- | --- |
| Effect | Estimates | *t* | *P* |
| Intercept | 0.199 ± 0.097 | 2.06 | 0.0415 |
| **Control Variables** | | | |
| Girls contrasted with boys | -0.096 ± 0.076 | -1.27 | 0.2056 |
| Mixed race contrasted with White | -0.149 ± 0.100 | -1.50 | 0.1366 |
| Black contrasted with White | 0.018 ± 0.181 | 0.10 | 0.9228 |
| Asian contrasted with White | 0.280 ± 0.162 | 1.73 | 0.0865 |
| Kindergarten mathematics achievement | 0.061 ± 0.047 | 1.29 | 0.1982 |
| Seventh grade mathematics achievement | 0.388 ± 0.048 | 8.04 | 0.0001 |
| Kindergarten reading achievement | -0.026 ± 0.053 | -0.49 | 0.6238 |
| Number processing speed | 0.023 ± 0.043 | 0.54 | 0.5883 |
| **Cognitive and In-Class Predictors** | | | |
| Intelligence | 0.064 ± 0.055 | 1.18 | 0.2414 |
| First grade phonological loop | -0.043 ± 0.059 | -0.72 | 0.4733 |
| First grade visuospatial sketch pad | -0.044 ± 0.046 | -0.94 | 0.3463 |
| First grade central executive | -0.058 ± 0.054 | -1.07 | 0.2842 |
| Fifth grade phonological loop | -0.006 ± 0.050 | -0.12 | 0.9011 |
| Fifth grade visuospatial sketch pad | 0.013 ± 0.046 | 0.29 | 0.7694 |
| Fifth grade central executive | 0.091 ± 0.050 | 1.81 | 0.0722 |
| In-class attentive behavior | 0.148 ± 0.048 | 3.08 | 0.0025 |
| **First Grade Mathematical Cognition Measures** | | | |
| Counting Competence | 0.050 ± 0.043 | 1.16 | 0.2483 |
| Number System Knowledge | 0.195 ± 0.060 | 3.25 | 0.0014 |

**Table S6 Ordinary Least Squares Estimates (± standard errors) for Prediction of Seventh Grade Mathematics Achievement Controlling for Numeracy.** *R*^2^ = .70, *F*_29,150_ = 12.18, *p*< .0001. The school site contrast is not shown and was not significant (*p* = .69).

|  | **Prediction of Mathematics Achievement** | | |
| --- | --- | --- | --- |
| Effect | Estimates | *t* | *P* |
| Intercept | -0.067 ± 0.139 | -0.48 | 0.6288 |
| **Control Variables** | | | |
| Girls contrasted with boys | 0.082 ± 0.107 | 0.76 | 0.4485 |
| Mixed race contrasted with White | 0.211 ± 0.141 | 1.50 | 0.1364 |
| Black contrasted with White | -0.010 ± 0.256 | -0.04 | 0.9686 |
| Asian contrasted with White | 0.195 ± 0.231 | 0.84 | 0.3997 |
| Kindergarten mathematics achievement | 0.038 ± 0.067 | 0.57 | 0.5711 |
| Seventh grade numeracy score | 0.776 ± 0.097 | 8.04 | 0.0001 |
| Kindergarten reading achievement | 0.070 ± 0.074 | 0.94 | 0.3465 |
| Number processing speed | -0.065 ± 0.060 | -1.08 | 0.2811 |
| **Cognitive and In-Class Predictors** | | | |
| Intelligence | 0.023 ± 0.078 | 0.30 | 0.7648 |
| First grade phonological loop | 0.026 ± 0.084 | 0.30 | 0.7619 |
| First grade visuospatial sketch pad | -0.026 ± 0.066 | -0.39 | 0.6970 |
| First grade central executive | 0.194 ± 0.076 | 2.56 | 0.0115 |
| Fifth grade phonological loop | 0.017 ± 0.071 | 0.24 | 0.8114 |
| Fifth grade visuospatial sketch pad | 0.043 ± 0.065 | 0.67 | 0.5036 |
| Fifth grade central executive | -0.000 ± 0.072 | -0.00 | 0.9974 |
| In-class attentive behavior | -0.079 ± 0.070 | -1.14 | 0.2577 |
| **First Grade Mathematical Cognition Measures** | | | |
| Counting Competence | -0.051 ± 0.061 | -0.82 | 0.4109 |
| Number System Knowledge | 0.014 ± 0.088 | 0.16 | 0.8760 |

**Growth in Number System Knowledge**

The measures that defined the Number System Knowledge factor were administered in first through fifth grade, inclusive. In sixth grade, the number line measure was modified from a 0-to-100 range to a 0-to-1000 range and thus number system knowledge scores beyond fifth grade are not directly comparable to those up to and including fifth grade. A principle components factor analysis, with promax rotation [3,37], confirmed that the four variables defined the same Number System Knowledge factor identified for first grade in second to fifth grade, inclusive (Eigenvalues > 1.76, factor loaders > |.54|).

To make each measure comparable to the others and across grades, the associated scores were defined as the percentage of maximum possible performance; specifically, for simple addition (number of problems correctly retrieved/14), for complex addition (number of problems correctly solved with decomposition/6), for Number Sets (RT adjusted d-prime score/maximum score achieved in fifth grade across all children), and number line [1 – (mean error/50)]. Fifty was chosen for the latter, because random placements would, on average, result in mean errors of 50 on the 0-to-100 number line. A child making random placements would thus have a score of 1-1, or 0 percent. The most accurate child in our study had a mean error of 1.75 in fifth grade, resulting in a score of 0.965.

The three groups shown in Figure 1 (main text) represent scores in the bottom (Low), two middle (Average), and top (High) quartiles on the seventh grade functional numeracy measure. A mixed model, with random intercept and random linear and non-linear grade effects, was used to assess group differences in start point and growth, controlling for the control variables in Table 2 (main text), intelligence, first grade working memory, and in-class attentive behavior. All groups differed from one another on start point (low vs. high, *p*=.0019; average vs. high, *p*=.0225, low vs. average, *p*=.0803).

With respect to growth, both the linear and non-linear effects were significant comparing the low and high groups (*p*s=.0011, .0099) and the low and average groups (*p*s=.0002, 0.0187), but neither effect was significant comparing the average and high groups (*p*s>.5275).

These effects were due to the group differences in rate of improvement from first to second grade. From second to fifth grade, the rate of improvement in number systems knowledge did not differ comparing any of the groups (*p*s>. 0.3526).

**References**

1. Pickering, S. and Gathercole, S. (2001). Working Memory Test Battery for Children (WMTB-C) Manual. London: Psychological Corporation Ltd.

2. Baddeley, A. D., & Hitch, G. J. (1974). Working memory. In G. H. Bower (Ed.), The psychology of learning and motivation: Advances in research and theory (Vol. 8, pp. 47-90). New York: Academic Press.

3. Bynner J. (1997). Basic skills in adolescents’ occupational preparation. Career Development

Quarterly 45: 305-321.

4. Hanushek, E. H., & Woessmann, L. (2008). The role of cognitive skills in economic

development. Journal of Economic Literature 46: 607–668

5. Murnane, R. J., Willett, J. B., Braatz, M. J., & Duhaldeborde, Y. (2001). Do different dimensions of male high school students’ skills predict labor market success a decade later? Evidence from the NLSY. Economics of Education Review 20: 311–20.

6. Rivera-Batiz, F. (1992). Quantitative literacy and the likelihood of employment among young adults in the United States. Journal of Human Resources 27: 313-328.

7. Berlin, G., & Sum, G. (1988). Toward a more perfect union: Basic skills, poor families, and our economic future. New York: Ford Foundation.

8. Kirsch, I. S., & Jungeblut, A. (1986). Literacy: Profiles of America’s young adults. Princeton, NJ: Educational Testing Service. Accessed at <http://www.ets.org/research/policy_research_reports/16-pl-02>.

9. Hudson, C., Price, D., & Gross, J. (2009). The long-term costs of numeracy difficulties. London, UK: Every Child a Chance Trust.

10. Imbo, I., & Vandierendonck, A. (2007). The development of strategy use in elementary

school children: Working memory and individual differences. Journal of Experimental Child Psychology 96: 284-309.

11. Denckla, M. B., & Rudel, R. (1976). Rapid automatized naming (RAN): Dyslexia differentiated from other learning disabilities. Neuropsychologia 14: 471-479.

12. Mazzocco, M. M. M., & Myers, G. (2003) Complexities in identifying and defining

mathematics learning disability in the primary school-age years. Annals of Dyslexia 53: 218-253.

13. Raven, J. C., Court, J. H., & Raven, J. (1993). Manual for Raven’s Progressive Matrices and

Vocabulary Scales. London: H. K. Lewis & Co.

14. Wechsler, D. (1999). Wechsler Abbreviated Scale of Intelligence. San Antonio, TX: PsychCorp,

Harcourt Assessment, Inc.

15. SAS Institute (2004). Statistical analysis system 7.5. Cary, NC: Author.
